# Effect of blackberry anthocyanins and its combination with tea polyphenols on the oxidative stability of lard and olive oil

**DOI:** 10.3389/fnut.2023.1286209

**Published:** 2023-11-29

**Authors:** Han Chen, Hui-fang Zhao, Xiu-hua Meng, Jian Chen, Wen-long Wu, Wei-lin Li, Han Lü

**Affiliations:** ^1^Jiangsu Key Laboratory for the Research and Utilization of Plant Resources, Institute of Botany, Jiangsu Province and Chinese Academy of Sciences (Nanjing Botanical Garden Mem. Sun Yat-Sen), Nanjing, China; ^2^Department of Food Science and Technology, College of Light Industry and Food Engineering, Nanjing Forestry University, Nanjing, China; ^3^Co-Innovation Center for Sustainable Forestry in Southern China, Forestry College, Nanjing Forestry University, Nanjing, China

**Keywords:** antioxidant, blackberry anthocyanins, composite antioxidants, lard, olive oil, tea polyphenols

## Abstract

To investigate the protective effect of blackberry anthocyanins (BA), tea polyphenols (TP), and their binary mixture on the oxidative stability of edible oils during storage, BA, TP, and their binary mixture were added to lard and olive oil. The changes in peroxide value (PV), thiobarbituric acid reactive substances (TBARS), acid value (AV), and scavenging capacity of DPPH and ABTS^•+^ of oil samples were evaluated during accelerated storage. BA were found to have a remarkable capability to enhance antioxidant properties, delay lipid oxidation, and inhibit the deterioration both of lard and olive oil at high-temperature processes. Furthermore, the antioxidant synergistic effect of BA and TP was found both in lard and olive oil for the first time. All these results suggested that BA and its combination with TP might possess the potential value to protect the quality of edible oils.

## Introduction

Lipid oxidation, one of the major factors in the deterioration of edible oils during process and storage, may result in changes in the major quality control parameters such as color, flavor, and nutritive value and further in the shelf-life reduction (1). Moreover, it leads to the formation of various potentially toxic products that may increase the risk of cancer, obesity, aging, and cardiovascular diseases (2).

Antioxidants have the capability of decelerating the oxidation rate of lipids, which effectively prolongs the shelf-life of edible oils (3). Many synthetic antioxidants such as butylated hydroxytoluene (BHT), butylated hydroxyanisole (BHA), and tertiarybutyl hydroxyquinone (TBHQ) are widely added to oils to increase oxidative stability (4). However, they have been well documented to have potential health risks resulting from their toxicity and carcinogenicity (5). Research studies indicated that natural antioxidants, which are beneficial to human health, have the ability to retard or prevent lipid oxidation (6). Consequently, there is a growing interest in the search for effective and safe natural antioxidants.

Many reports have confirmed that plant extracts rich in polyphenols are important candidates for alternatives to synthetic antioxidants. Blackberry, a worldwide popular fruit, is commonly utilized in processed products, such as jam, jelly, and juice or used as an ingredient in various food products (7). Blackberry contains abundant polyphenols, particularly anthocyanins, which were reported to exhibit strong antioxidant properties (8). In terms of food additives, the crude anthocyanin extract of blackberry could improve the oxidative stability of rapeseed oil (9).

Tea polyphenols (TP) is the name of polyphenols extracted from tea. Most TP are flavonols, namely catechins, including epicatechin (EC), epigallocatechin (EGC), epicatechin gallate (ECG), and epigallocatechin gallate (EGCG) (10). TP have been intensively studied and demonstrated multiple health-promoting properties (11). Several studies confirmed that TP possess very strong antioxidant activities (12–14). In addition, TP receive wide attention as efficient food antioxidants for their protective effects on the physicochemical properties and sensory characteristics of foods (15). The addition of TP exhibited an excellent antioxidant effect on the extraction process of Antarctic krill oil (16), and in another research, TP inhibited the deterioration of acid value, peroxide value, anisidine value, and color of rapeseed oil during the frying process (17).

Some research studies reveal that the addition of combinations of antioxidants has a more pronounced effect than that of the individual one, which is termed antioxidant synergism. A study on the combination of TP and α-tocopherol has shown synergistic antioxidant effects on different lipid systems based on high linoleic sunflower oil (18). However, the combinations of TP, catechin, or epicatechin with α-tocopherol resulted in antioxidant antagonistic effects (18, 19). It is not clear that the combinations of blackberry anthocyanins and TP may result in antioxidant synergism or antagonistic effects.

In this study, two types of edible oils–lard (animal fat) and olive oil (vegetable original oil) were selected as test materials. Single blackberry anthocyanins and TP or their binary mixture were added to two types of oils. The effect of these natural antioxidants on the oxidative stability of two types of oils was investigated in an accelerated storage experiment. The combination of blackberry anthocyanins and TP was also evaluated for their antioxidative synergistic effect. This study will indicate these natural antioxidants for their properties in protecting the quality of animal fat and vegetable original oil. The results may provide the potential of BA or the combination of BA and TP to replace the synthetic antioxidants in edible oils.

## Materials and methods

### Chemical and reagents

Acetonitrile, methanol, and formic acid (HPLC grade) were obtained from Tedia Co. Inc. (Fairfield, OH, USA). Ethanol and phosphoric acid were purchased from Sinopharm Chemical Reagent Co. Ltd. (Shanghai, China). Tea polyphenol (TP) was purchased from Yuanye Bio-Technology Co., Ltd. (Shanghai, China). Butylated hydroxytoluene (BHT), 2,2-diphenyl-1-picrylhydrazyl (DPPH), and 2,2'-azinobis-3-ethylbenzothiazoline-6-sulfonic acid (ABTS) were obtained from Sinopharm Chemical Reagent Co., Ltd. (Shanghai, China). Cyanidin-3-*O*-glucoside was isolated from blackberry in our previous study. Lard without additives was purchased from a local market (Nanjing, China). Virgin olive oil without additives and antioxidants (acid value < 0.5%) was obtained from Elbek SA (Spain).

### Preparation of blackberry anthocyanins

Fresh blackberry (*Rubus fruticosus* Pollich) was harvested from the Baima plantation (Nanjing, Jiangsu Province, China). Blackberry was homogenized by a blender and extracted by ultra-sonication with 70% ethanol containing 0.1% formic acid (v/v). The ethanol solution was concentrated under vacuum and centrifugated at 4°C at 10,000 r/min for 10 min. The supernatant was purified with AB-8 macroporous resin. After elution with pure water, the anthocyanin was eluted with 75% ethanol containing 0.1% formic acid. The extract was concentrated under vacuum and lyophilized to obtain a homogenous powder, which was used in subsequent experiments as blackberry anthocyanin (BA).

### Detection of blackberry anthocyanins

HPLC was carried out on a Dionex Ultimate 3000 HPLC system (Thermo Fisher Scientific Inc., Germany), equipped with a DAD detector. A YMC- Pack ODS-AQ (4.6× 250 mm, 5μm, YMC CO., LTD., Japan) column was used. The mobile phase consisted of acetonitrile (A) and water (containing 0.34% phosphoric acid) (B) with linear elution (0–15 min, 90–85% B; 15–30 min, 85–70% B; 30–35 min, 70–60% B; 35–36 min, 60–10% B; 36–41 min, 10% B). The flow rate was 1 ml·min^−1^, and anthocyanin was detected at 520 nm. Anthocyanin was identified by matching the peak retention time with the reference substance. The total content of anthocyanin was calculated as mg of cyanidin-3-*O*-glucoside per gram of extract sample.

### Preparation of test samples

BA, TP, and a mixture of TP and BA (1:1, m/m) (BA+TP) were added to lard (preheated to 75°C) and olive oil to achieve the concentration of 200 ppm as test oil samples (namely BA, TP, and BA+TP, respectively). BHT was added to oil samples with a final concentration of 100 ppm as positive control samples (named BHT). The control samples were prepared with the same oil without any antioxidants.

### Accelerated storage experiment

The oil samples were separately placed in conical flasks and stored in a baking oven at the temperature of 60°C for 14 days.

### Analysis of antioxidant activity

Changes in antioxidant activity of various oil samples during heat accelerated storage were evaluated by DPPH radical scavenging assay and ABTS radical scavenging assay.

DPPH assay was carried out according to the reported method (20). In total, 80 μl of oil samples were dissolved in 2.00 ml of ethanol as test solutions. Overall, 1.0 ml of test solution was mixed with 1.0 ml of 0.1 mM DPPH ethanolic solution. After 30 min, the absorbance was measured at 515 nm by a microplate reader. A blank sample containing 100 μl of methanol in the DPPH solution was measured. The antioxidant capacity was expressed as Trolox equivalent per kilogram of oil (mg TE/kg).

ABTS^•+^ (ABTS radical cation) was obtained from a reaction of 2 mM ABTS aqueous solution with 0.7 mM potassium persulfate and then the mixture was kept in a dark environment at room temperature for 12 h as described previously (21). ABTS reaction solution was diluted to 0.70 ± 0.02 at an absorbance of 734 nm with methanol. In total, 15 μl of oil sample was dissolved in 6 ml ABTS reaction solution. After 7 min, the absorbance at 515 nm was measured. A blank sample containing ABTS reaction solution was measured. Antioxidant capacity was expressed as Trolox equivalent per kilogram of oil (mg TE/kg).

### Peroxide value

Peroxide value (PV) was evaluated according to the official method 965.33 of AOAC (22) with slight modification (23). Oil samples (2.00 g) were dissolved in 30 ml of acetic acid–chloroform (3:2, v/v) solution, and 1 ml of saturated potassium iodide (KI) was added. The mixture was shaken sufficiently and kept in a dark environment for 5 min. After adding 75 ml of distilled water, the mixture was titrated with 0.01 M sodium thiosulfate (Na_2_S_2_O_3_) until the yellow color disappeared. Then, 0.5 ml of 1% starch solution was added, and the titration was continued until the blue color disappeared. The blank was measured under a similar condition. The PV of each sample was determined according to the measurement in triplicate, and PV (mEq/kg) was calculated using an equation as follows:


$$\begin{array}{l} \text{PV }\left(\text{mEq}/\text{kg}\right)=\text{C }\times \;\left(\text{V}-{\text{V}}_{0}\right)\;\times \;12.69\;\times \;78.8/\text{W} \end{array}$$

where C is the concentration of Na_2_S_2_O_3_ (mol/L), V is the volume of Na_2_S_2_O_3_ consumed in the titration process of the sample (ml), V_0_ is the volume of Na_2_S_2_O_3_ consumed in the titration process of blank (ml), and W is the weight of the olive oil or lard (g).

### Acid value

Acid value (AV) was used to measure the rancidity of oil samples according to the official method 969.17 of AOAC (22) with some slight modifications. Olive oil samples (2.00 g) or lard samples (2.00 g) were added to a 250 ml flask. Then, 30 ml of isopropyl alcohol–petroleum ether (1:1, v/v) solution was added to the flask above, and the mixture was shaken sufficiently until the oil samples dissolved completely. After the addition of 2–3 drops of phenolphthalein indicator, the mixture was titrated with 0.1 M potassium hydroxide (KOH) until a reddish tinge appeared without fading. The determination for AV of oil samples was calculated according to the equation:


$$\begin{array}{l} \text{AV}\left(\text{g}/\text{g}\right)=\left(\text{V}-{\text{V}}_{0}\right)\;\times \;0.1\text{M }\times \;56.1/\text{W} \end{array}$$

where V is the volume of KOH consumed in the titration process of the sample (ml), V_0_ is the volume of KOH consumed in the titration process of blank (ml), M is the molarity of KOH, W is the weight of the olive oil or lard (g).

### Thiobarbituric acid reactive substance value

The thiobarbituric acid reactive substance (TBARS) value of the oil sample was evaluated according to the reported method (24). In total, 1 ml of olive oil or lard was dissolved in 2 ml of 20 mM 2-thiobarbituric acid (TBA) solution containing 15% trichloroacetic acid (TCA) (w/v). The mixture was incubated in boiling water for 15 min until a pink color appeared. Subsequently, the mixture was cooled under running tap water followed by centrifugation for 15 min at 4,500 rpm at 25°C. The blank of this assay was distilled water under the same conditions. The absorbance of the supernatant was measured at 532 nm. 1,1,3,3-Tetraethoxypropane (TEP) was used to prepare a standard curve for malondialdehyde (MDA), where TBARS value was expressed as MDA equivalent mg per kg of sample.

### Statistical analysis

Statistical analysis was performed using GraphPad Prism version 8 (GraphPad Software, Inc., CA, USA). Each independent analysis was performed in triplicate (*n* = 3) for which the results were expressed as mean ± standard deviation. Collected data were calculated using one-way ANOVA followed by Duncan's test for the comparison of differences between means of parameters at the 5% significance level. Differences were considered significant at *p* < 0.05.

## Results and discussion

### Content of blackberry anthocyanins

The qualitative and quantitative analyses of anthocyanins in blackberry anthocyanin (BA) extract were carried out using an HPLC method. The extract mainly contains four anthocyanins: cyanidin-3-*O*-glucoside, cyanidin-3-*O*-arabinoside, cyanidin-3-*O*-(6″-malonylglucoside), and cyanidin-3-*O*-(6″-dioxalylglucoside). The dominative anthocyanin in BA was cyanidin-3-*O*-glucoside. The total content of anthocyanins was 661.22 mg/g (mg cyanidin-3-*O*-glucoside per gram of extract sample).

### Comparison of the anti-lipid-oxidant efficiency of different antioxidants

**Figure 1:**
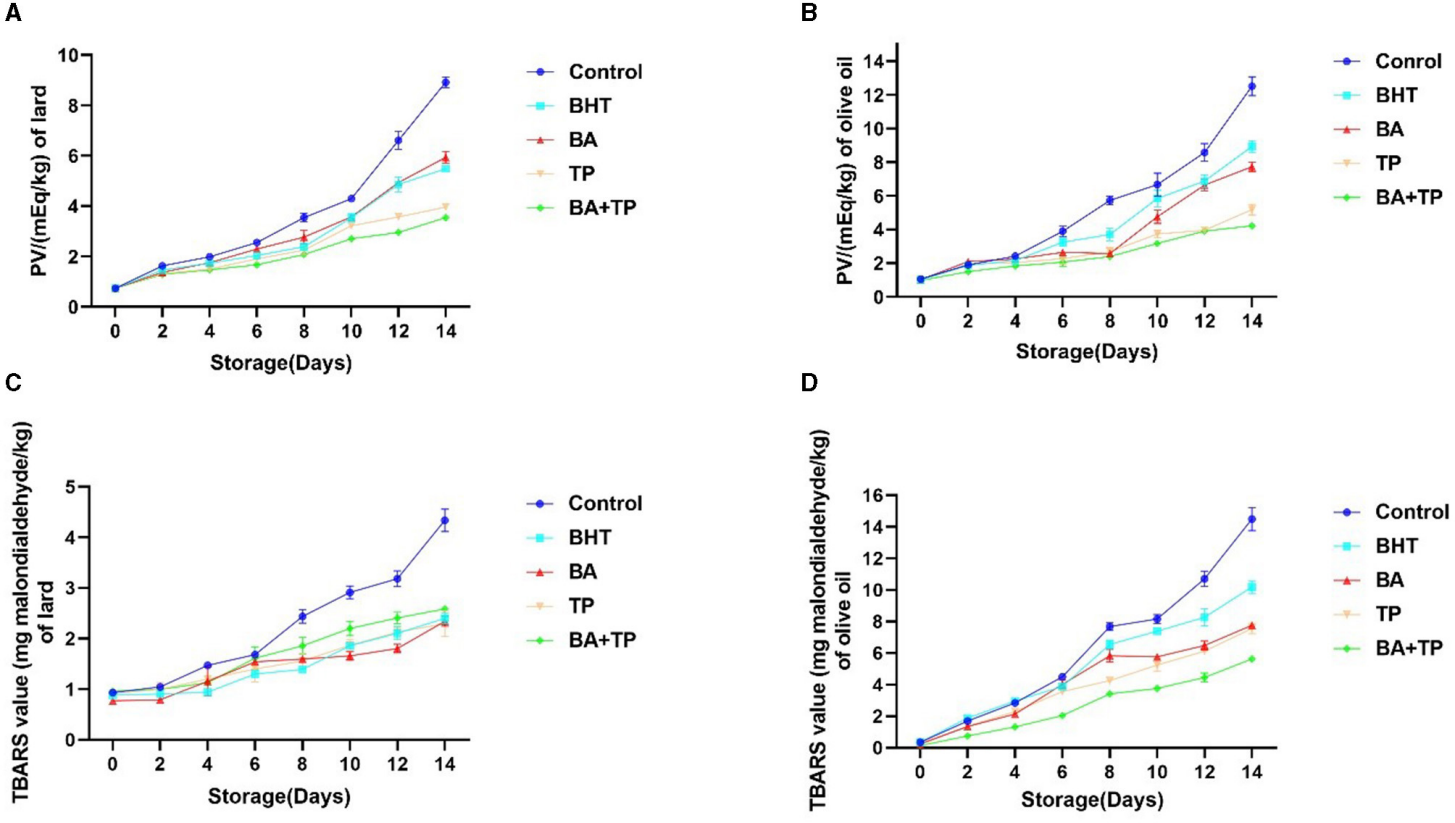
Changes in peroxide value (PV) (mEq/kg) and TBARS value (mg MDA/kg) of lard **(A, C)** and olive samples **(B, D)** without oxidant (control) or addition of different antioxidants: 200 ppm of blackberry anthocyanins (BA), 200 ppm of tea polyphenols (TP), the mixture of 100 ppm of BA and 100 ppm TP (BA+TP) and 100 ppm of butylated hydroxytoluene (BHT) during 14 days of accelerated storage at 60°C.

The lipid oxidation products are categorized into primary and secondary products. The peroxide value (PV) analysis is performed to monitor the primary oxidation products in this research (Figures 1A, B). A 14-day heated storage significantly elevated the PV of the control lard sample. The addition of BHT, BA, TP, and a mixture of BA and TP (BA + TP) significantly lowered the PV (*p* < 0.001) compared with the control sample, indicating that these antioxidants could retard the primary oxidation of lard. The order of inhibitory ability was as follows: BA + TP >TP > BHT>BA; the mixture of antioxidant BA + TP had the most excellent inhibitory effect. Compared with lard, the PV of olive oil had a higher rate of increase, and the PV of control olive oil increased by 12 times after 14-day heated storage. All antioxidants significantly prevent the primary oxidation of olive oil compared with the control sample (*p* < 0.001). The order of inhibitory ability was as follows: BA + TP >TP > BA >BHT; BA + TP also showed greater antioxidant efficiency than the other antioxidants.

To evaluate the formation of secondary oxidation products during the accelerated storage process, TBARS values of lard and olive oil samples were determined (Figures 1C, D). After 14 days of accelerated storage, the TBARS value of the control lard sample was four times larger than that of 0 day. The addition of BHT, BA, TP, and BA + TP significantly declined the TBARS of lard samples compared with the control sample (*p* < 0.001), which indicated that these antioxidants could remarkably retard the secondary oxidation of lard. However, there were no significant differences in TBARS value among samples added with different antioxidants (*p* > 0.05). Similarly, a remarkable surge in TBARS value was found in an olive oil control sample after 14-day heated storage. The addition of antioxidants significantly declined the TBARS values compared with the olive oil control sample (*p* < 0.001). The order of inhibitory ability was as follows: BA + TP > BA >TP >BHT, and BA + TP also showed stronger antioxidant efficiency than other antioxidants.

Lipid class, fatty acid profile, and composition are the crucial factors of the susceptibility of lipid oxidation. In addition, some external factors such as temperature, light, activators, and inhibitors may influence lipid oxidation and directly affect the quality of the final product (25). The 14-day heated storage significantly elevated the PV and TBARS values of lard and olive oil, indicating that high temperate may accelerate the lipid oxidation process in oil samples. Compared with lard, olive oil had a faster rate of increase in PV and TBARS values. Olive oil possesses a high amount of unsaturated fatty acids, such as oleic acid, linoleic acid, or palmitic acid. Compared with olive oil, lard contains more saturated fatty acids such as palmitic acid and stearic acids and less linoleic acids (26). The higher content of unsaturated fatty acids may be related to the faster rate of lipid oxidation. It was reported that the addition of TP could reduce the formation of peroxides and secondary oxidation products of krill oil during the extraction process (16), and anthocyanin extracts of blackberry could decrease the PV and TBARS values of rapeseed oil during 14 days of storage at 40°C (9). Our study confirmed the anti-lipid-oxidant capacity of TP and BA in edible oils. It is interesting that the mixture of BA and TP exhibited a stronger antioxidant effect than a single BA or TP, which suggested a synergistic effect.

### Comparison of antioxidant capacity by determining the scavenging capacity of DPPH-free radicals and ABTS cation radicals

To further compare the influence of different antioxidants on the antioxidant capacity of oil samples, the scavenging capacity of DPPH- and ABTS-free radicals was carried out to evaluate the antioxidant capacity of different oil samples.

**Figure 2:**
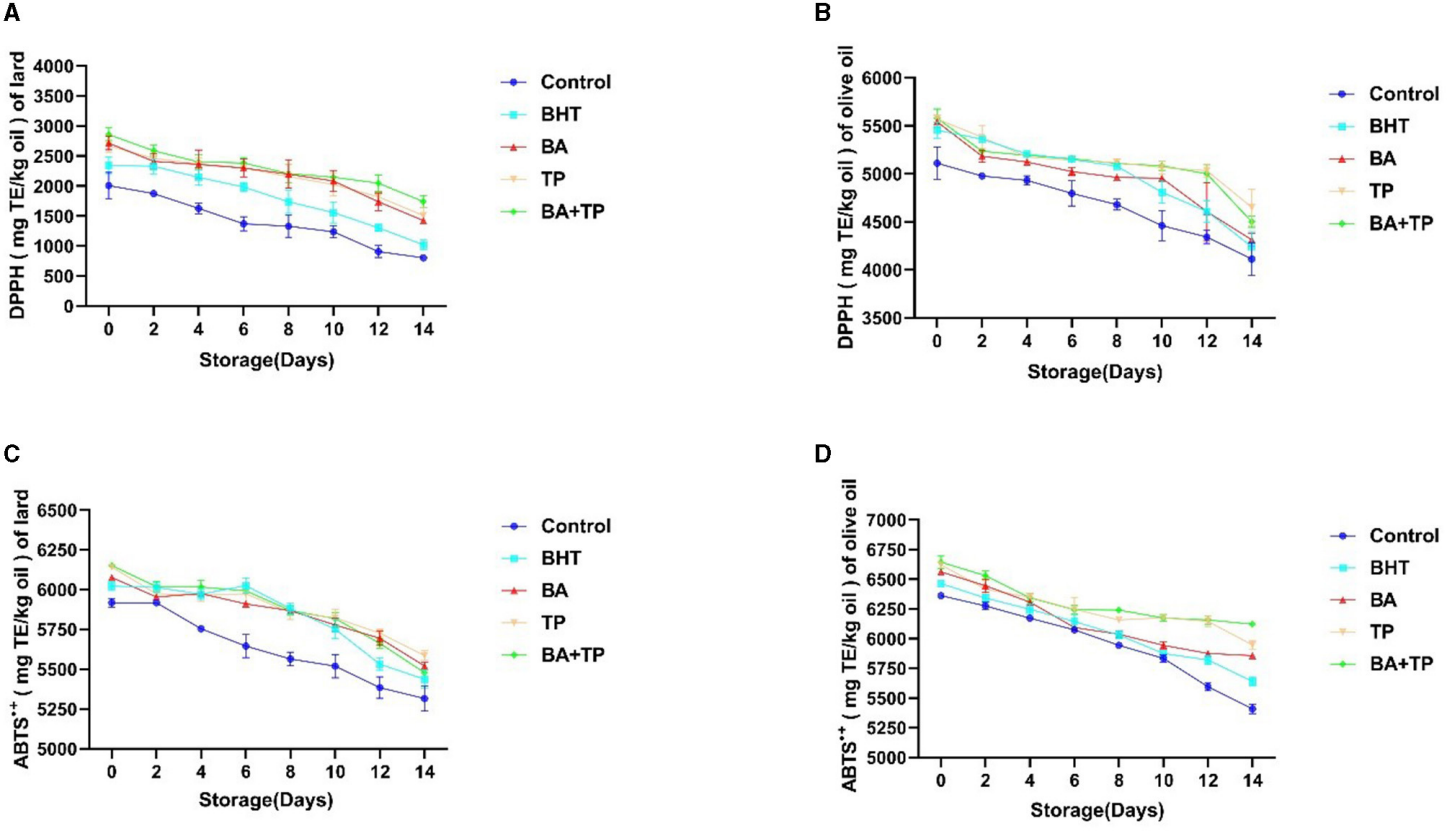
Changes in antioxidant capacity performed by DPPH and ABTS^•+^ scavenging assay of lard **(A, C)** and olive samples **(B, D)** without oxidant (control) or addition of different antioxidants: 200 ppm of blackberry anthocyanins (BA), 200 ppm of tea polyphenols (TP), the mixture of 100 ppm of BA, 100 ppm of TP (BA+TP), and 100 ppm of butylated hydroxytoluene (BHT) during 14 days of accelerated storage at 60°C.

As shown in Figure 2, the scavenging capacity of DPPH-free radicals of all the oil samples gradually declined in the heated storage process. The addition of test antioxidants significantly enhanced the scavenging capacity of DPPH of lard and olive oil in 0 day (*p* < 0.001) and delayed the decreasing trends of scavenging capacity. The order of scavenging capacity of DPPH of test lard samples was as follows: BA + TP >TP ≈ BA >BHT; however, there was no significant difference in scavenging capacity of DPPH among the olive oil samples added with different antioxidants. Similarly, the addition of antioxidants elevated the scavenging capacity and delayed the significant downward trends of ABTS of lard and olive oil. The order of scavenging capacity of ABTS of test samples added with different antioxidants was as follows: TP > BA > BA + TP > BHT (lard) and BA + TP > TP > BA > BHT (olive oil).

Previous studies exhibited that heat can reduce the oxidative stability of olive oil (27). In this study, the scavenging capacity of free radicals of olive oil and lard remarkably declined after 14 days of heated storage, indicating the attenuation of antioxidant capacity. The addition of test antioxidants can prevent the decay of the antioxidant capacity of both olive oil and lard. Similar to the result of the lipid oxidation assay, both BA and TP demonstrated stronger antioxidant capacity than BHT, in addition to the mixture of BA and TP exhibited a synergistic effect as well. Virgin olive oil contains abundant phenolic compounds represented by lipophilic phenols such as tocopherols and hydrophilic phenols including hydroxytyrosol, phenolic acids (i.e., benzoic acid, cinnamic acid, and their derivatives), lignans, and secoiridoids. These polyphenols can work as antioxidants to inhibit oxidation in oil (28). In this study, the olive oil sample showed a higher scavenging capacity of DPPH and ABTS than the lard sample, which may be related to the antioxidant activity of phenolic compounds in olive oil. Additionally, the abundant anthocyanins in BA and catechins in TP may interact with the endogenous antioxidant phenolic compounds in olive oil and depress the decay of its antioxidant capacity.

### Effect of different antioxidants on acid value

**Figure 3:**
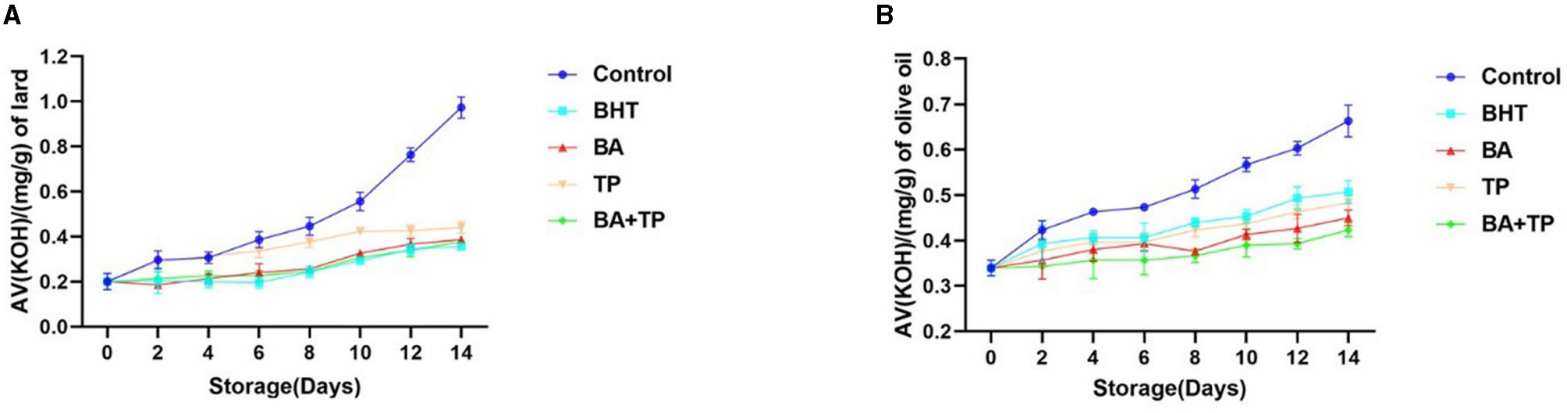
Changes in the acid value (AV) (mg/g) of lard samples **(A)** and olive oil samples **(B)** without oxidant (control) or addition of different antioxidants: 200 ppm of blackberry anthocyanins (BA), 200 ppm of tea polyphenols (TP), the mixture of 100 ppm of BA, 100 ppm of TP (BA+TP), and 100 ppm of butylated hydroxytoluene (BHT) during 14 days of accelerated storage at 60°C.

The acid value (AV) is one of the most important parameters to reflect the quality of edible oil. To evaluate the effect of different antioxidants on the quality of oil samples, an analysis of AV is carried out in this research. The changes in AV during the accelerated storage process are shown in Figure 3. After 14 days of accelerated storage, the AV of the control lard sample remarkably increased to a level that was almost five times the value of that on 0 day, while all the antioxidants significantly depressed the rising trend. There were no significant differences in the inhibitory ability among BA, BA+TP, and BHT, while all of them showed higher efficiency than TP. In the olive oil sample, 14-day accelerated storage also induced a significant increase in the AV value of the control sample. The addition of oxidants significantly prevented the increasing trend. The order of inhibitory efficiency was as follows: BA + TP > BA >TP > BHT (*p* < 0.05), and BA + TP exhibited a stronger inhibitory ability than other antioxidants.

AV is the indicator of free fatty acid content in oil, and the lower AV implies better freshness of the oil (29). An increasing acid value also correlates with a decrease in the nutritional value of edible oil, and long-term consumption of high AV edible oil may be harmful to human health (30). Previous studies showed that the addition of TP could effectively inhibit the increase in AV in rapeseed oil during the frying process (17). In our study, the addition of BA, TP, and BA + TP significantly inhibited the increasing trend of AV both in olive oil and lard, which suggested that these natural antioxidants can prevent the deterioration and enhance the stability of edible oils. Similarly, the mixture of BA and TP exhibited a more pronounced inhibitory effect than the individual sample, which further confirmed the synergism between BA and TP.

## Conclusion

In the present study, blackberry anthocyanins (BA) and its combination with tea polyphenols (TP) were found to have a remarkable capability to inhibit the deterioration of lard (animal fat) and olive oil (vegetable oil) during the accelerated storage experiment at 60°C. These natural antioxidants enhanced the antioxidant properties and delayed the lipid oxidation of edible oils at high-temperature processes. Furthermore, the antioxidant synergistic effect of BA and TP in edible oils was found for the first time. All these results suggested that BA and its binary mixture with TP might possess the potential value to protect the quality of edible oils, and they may be expected to be the substitutes for synthetic antioxidants in edible oils. However, their influence on the oxidative stability and some other qualitative indexes of oil such as fatty acid composition during different storage conditions including long-term room temperature storage need to be further studied to predict their effects on the quality and shelf-life of edible oils.

## Data availability statement

The original contributions presented in the study are included in the article/supplementary material, further inquiries can be directed to the corresponding author.

## Author contributions

HC: Formal analysis, Investigation, Writing – original draft. H-fZ: Project administration, Writing – review & editing, Resources. X-hM: Writing – review & editing, Data curation, Investigation. JC: Data curation, Writing – review & editing, Methodology. W-lW: Writing – review & editing, Formal analysis. W-lL: Writing – review & editing, Data curation. HL: Writing – review & editing, Investigation, Project administration, Writing – original draft.
